# Everyday Memory in Healthy Aging: Porous but Not Distorted

**DOI:** 10.3389/fnagi.2019.00153

**Published:** 2019-06-25

**Authors:** Karolina Sejunaite, Claudia Lanza, Matthias W. Riepe

**Affiliations:** Department of Psychiatry and Psychotherapy II, Division of Mental Health & Old Age Psychiatry, University of Ulm, Ulm, Germany

**Keywords:** memory, recognition, false alarms, daily life, neuropsychology, healthy aging

## Abstract

Most studies targeting age-association of memory functions report a decline in recognition hits and an increase in false alarms. The goal of the present study was to assess these findings in tasks with day-to-day relevance. We investigated healthy young (YA; age 26.90 ± 3.55 years) and old (OA; age 69.80 ± 5.85 years) adults. Participants were asked to watch six news and six commercials and complete a recognition task relating to the information presented in the videos. OA had a lower hit rate in both news and commercials compared to YA. However, the number of false alarms (FA) was the same in both age groups. Applying signal detection theory, we found age differences in discriminability for both news and commercials paradigm. The groups showed no differences in bias and both chose a liberal answering tendency. We interpret our finding as a result of complex recognition items in an ecologically valid task. Multi-feature items offer an advantage in correct rejection—it is enough to know that at least one feature of an item is false. This benefit does not extend to hits, where all features of an item need to be recognized. This indicates that recognition memory of naturalistic stimuli in OA is porous, but not distorted.

## Background

Aging goes along with a decline in various cognitive functions such as visual-constructive abilities, processing speed, and memory (Di Carlo et al., 2000; Park et al., 2002; MacDonald et al., 2004; Royall et al., 2005; Ardila, 2007). Changes in memory performance are not homogeneous and different aspects of memory change differently. Among memory deficits, episodic memory performance undergoes the most pronounced changes with increasing age (Souchay et al., 2000; Daselaar et al., 2003; Kramer et al., 2003; Nilsson, 2003; Salthouse, 2016). Concerning memory processes, literature suggests that the most drastic deterioration occurs in recall (Hultsch, 1969; Petersen et al., 1992; Hertzog et al., 2010), whereas recognition is only moderately affected (Jacoby, 1999; Ratcliff et al., 2004; Spaniol et al., 2006; Van Ocker et al., 2017).

Recognition errors comprise two kinds of errors, not recognizing studied items (misses), and falsely recognizing new unstudied items (false alarms, FA; Stanislaw and Todorov, 1999; Shapiro, 2014). A recent meta-analysis of 232 studies by Fraundorf et al. (2019) has reported that younger adults generally outperformed older adults (OA) in recognition tasks and that older adults showed a more liberal answering tendency resulting in an increase in false alarms. Interestingly, it was reported that age differences in recognition accuracy were largest for easy tasks. Furthermore, older adults memory showed an even greater detriment, when recognition task involved new combinations of already familiar items. The main effect of age on recognition memory persisted across different studies in spite of variability in learning material, emotional valance of the stimuli or retention interval, suggesting that recognition memory decline involves a general process operating across different modalities. An increase of erroneous memory content was also previously observed in different paradigms such as a recognition version of the Deese-Roediger-McDermott (DRM) procedure (Roediger and McDermott, 1995; Seamon et al., 2002), conceptual similarity (Paige et al., 2016), self-related information (Rosa et al., 2015), misinformation (Meade et al., 2012), and misattribution (Mitchell and Johnson, 2009).

While such paradigms are useful to test theoretical models and advance understanding of memory construct, it has been questioned if they can fully account for cognitive processes faced by healthy older and younger persons on a daily basis (Chaytor and Schmitter-Edgecombe, 2003). A recent study reported that DRM and other above mentioned tasks do not sufficiently predict memory outcomes and questioned the implications to use these paradigms to predict memory distortions in real-life situations (Patihis et al., 2018).

One way to bridge the gap between laboratory and ecologically valid paradigms would be to create assessments using verisimilitude approach, which refers to development of tests that are comprised of everyday cognitive tasks. Such instruments were developed for everyday skills such as attention (Robertson et al., 1994), executive function (Wilson et al., 1996) and some aspects of memory (Wilson et al., 2004, 2008). A study of memory for commercials reported that despite having less correct rejections than young adults (YA), older adults demonstrated better memory for emotionally meaningful content compared to neutral one (Fung and Carstensen, 2003). Another study by Mazurek et al. (2015) required healthy younger and older participants to hide objects in a room, which was later followed by a surprise memory task, where participants were asked to freely recall *what, where*, and *when* they had hidden. Results showed that older adults recalled significantly less full combinations of object, location and time, but memory for incomplete combinations (i.e., *what-where*, *what-when*, and *where-when*) did not differ between the two groups. Furthermore, the ability to bind the three features together varied significantly in the older adults group, with some individuals performing worse and some better than younger adults. The authors partly explained this finding through differences in memory strategies used by the participants—an ecologically valid task might make it easier for older adults to apply more efficient memory strategies, such as “mental travel,” which they rely upon in everyday life. The performance on the new memory task was predicted by the existing measures of episodic memory. Additionally and in contrast to existing memory measures, the performance on the new memory task was also predicted by the self-evaluation memory questionnaires. The authors suggested that such ecologically valid task could offer an advantage over conventional scales by picking up on very early signs of cognitive decline in older population.

Exposure to material frequently seen on TV, which often serves as a most common information acquisition source, has been studied previously. Daily exposure to material such as news or commercials influences our attitudes, preferences and decision making (Martin, 2003; Prior, 2003; Weitzer and Kubrin, 2004). For that reason, it is important to assess memory for this kind of information presentation across different age groups. Thus far, studies using real life paradigms of news or commercials in healthy populations focused primarily on brain activity (Frings et al., 2010), emotional salience (Fung and Carstensen, 2003) or pre-existing attitudes (Frenda et al., 2013) rather than false alarms. Although recognition memory using such everyday life material has already been investigated in the context of disorders directly affecting memory performance (Sejunaite et al., 2017, 2018), the pattern and causes of false alarms with healthy aging in everyday life remains vague.

Generally, the age-related decline in hits and the increase in false alarms are linked to changes in brain activity. Functional imaging studies reported an increased task-related activity in the left temporal, frontal, and posterior parietal cortex among older adults during memory tasks (Frings et al., 2010; Craik and Rose, 2012). This pattern was thought to reflect increased effort and decreased reliance on automatic effortless processing in older subjects compared to young adults. Two brain regions that seem to be affected the most by aging were medial temporal and prefrontal cortex (Buckner, 2004; Butler et al., 2004; Hedden and Gabrieli, 2004; Raz et al., 2005; Meade et al., 2012). They were associated with increased susceptibility to lure items in recognition (Plancher et al., 2009; Fandakova et al., 2013a) as well as increased misattribution of memory sources (Craik et al., 1990; Chan and McDermott, 2007; Fandakova et al., 2013b) respectively. Aging-related hippocampal volume reduction puts a strain on effective memory process requiring other cortical areas, such as prefrontal cortex, to compensate for the deficits (Cabeza, 2002; Cabeza et al., 2002; Persson et al., 2006). In contrast, older adults that were able to maintain memory processing patterns akin to those in young adults were reported to have fewer false recognitions (Fandakova et al., 2015).

Further support for the involvement of decreased frontal and medial temporal functioning in false recognitions comes from studies that compared memory performance with neuropsychological correlates. Better performance in memory measures such as free and cued recall was shown to contribute to binding separate features into complex memories and increase the number of hits (Henkel et al., 1998; McCabe et al., 2009). Higher scores on frontal function measures such as verbal fluency, working memory, abstract reasoning and problem solving were associated with less false alarms (Roediger and Geraci, 2007; McCabe et al., 2009) and better evaluation of contextual features of memory traces (Henkel et al., 1998).

To account for this discrepancy in memory performance with age, multiple explanations were proposed (Johnson and Raye, 1998; Roediger et al., 2001; Devitt and Schacter, 2016). Although theories explaining age-related increase in false alarms are numerous, three of them are viewed as the most prominent to explain age-associated factors (Healey and Kahana, 2016). Associative deficit hypothesis (Naveh-Benjamin, 2000; Old and Naveh-Benjamin, 2008) postulates that older adults are selectively impaired in the ability to bind separate features together into meaningful memory traces. This association deficit persists even when memory for features themselves is intact (Chalfonte and Johnson, 1996). This hypothesis helps to explain the source monitoring deficits frequently reported in older adults and often viewed as a distinct false memory theory. Inhibitory deficit hypothesis (Hasher and Zacks, 1988; Healey and Kahana, 2016) argues that age-related increase in false alarms arises due to decreased ability to inhibit irrelevant information. This hypothesis helps to explain the effects of interference (Hamm and Hasher, 1992; May and Hasher, 1998; Manard et al., 2014) as well as effects of priming (Madden, 1986; Ikier et al., 2008) on memory. Last, according to cognitive slowing hypothesis (Salthouse, 1996a, b), aging is associated with a general reduction in most cognitive abilities (Ardila et al., 2000; Park et al., 2002; Darowski et al., 2008; Zahodne et al., 2011). According to this theory, a diminished performance results from an age-associated decrease of the speed of cognitive processing—controlling for speed explains up to 70% of variance in memory tasks (Aminoff et al., 2012).

Taken together the literature suggests that older persons are more prone to false alarms. The studies, however, mostly used experimental paradigms loosely related to day-to-day situations. The goal of the present study was to investigate recognition memory with ecologically valid material (news and commercials) in healthy aging. We hypothesized that older adults would have more false alarms in an ecologically valid memory paradigm than young adults. Furthermore, we hypothesized that age differences would be explained by age-associated decline of performance on other cognitive tasks such as measures of frontal function.

## Participants

A statistical power analysis was performed for sample size estimation, based on data from a similar recognition memory study for advertisements (Fung and Carstensen, 2003). Using the means and standard deviations for neutral material the calculated effect size *d* was 1.07. With an alpha = 0.05, and power = 0.90, the sample size estimated with this effect size is 16 per group for between-group comparisons. The present study is a follow-up study to two prior studies assessing false alarms in healthy older persons and older persons with depressive disorder or Alzheimer’s dementia (Sejunaite et al., 2017, 2018). The group of older persons in the present study was the same as in the prior studies. We, therefore, chose to use a similar sample size for the group with young healthy adults. The sample in the present study comprised a total of 21 young adults [YA; age 21–35 years; 26.90 ± 3.55 (Mean ± Standard Deviation); 13 females] and 20 older adults (OA; age 61–83 years; 69.80 ± 5.85; 10 females). Both, YA and OA consisted of volunteers recruited by local advertising to partake in a study on memory and aging. Central nervous system disorders such as neurodegenerative disorders and affective disorders as well as mental retardation and addictive behavior were ruled out by taking medical history. The exclusion criteria for Mini Mental State Examination (MMSE) and Montgomery-Asberg-Depression-Rating-Scale (MADRS) were <28 and >9, respectively.

## Materials and Methods

The present study was designed as a non-randomized, cross-sectional study. All participants were briefed about the study design. The neuropsychological assessment as well as experimental sessions took place at the Division of Mental Health and Old Age Psychiatry of Ulm University. The study received approval of the local ethics committee and was done in accordance with the local ethical standards of the Ulm University and the guidelines outlined in the Declaration of Helsinki (World Medical Association, 2013).

### Neuropsychological Assessments

#### Clinical Scales

##### Mini-Mental State Examination (MMSE; Folstein et al., 1975)

The MMSE is a widely used instrument to give an overview over global cognitive functioning. It comprises questions on orientation, registration, short-term memory, language use, comprehension, and basic motor skills. The score ranges from 0 to 30, with a score below 24 indicating a cognitive impairment.

##### Montgomery-Asberg-Depression-Rating-Scale (MADRS; Montgomery and Asberg, 1989)

The score in the MADRS reflects the affective state of the examinee as assessed by a health care professional and consists of 10 aspects to be evaluated: apparent sadness, communicated sadness, inner tension, sleep, appetite, concentration, impetus, callousness, pessimistic thoughts and suicidal ideation. Each of the aspects is given a score from 0 to 6 according to its severity. The total score ranges from 0 to 60. Scores 0–8 indicate no depression, 8–16 a mild, 16–24 a moderate, and 24 and higher a severe depression.

#### Neuropsychological Tests

##### Vocabulary Test (Wortschatztest, WST; Schmidt and Metzler, 1992)

In the Wortschatztest (WST), the examinee needs to find an actual word among five non-word distractors. The word list among which the actual word needs to be chosen increases in difficulty as the test progresses. The number of correct answers (maximum 40) is counted and the raw values are converted into IQ scores.

##### California Verbal Learning Test (CVLT; Niemann et al., 2008)

The California Verbal Learning Test (CVLT) is a verbal memory test, assessing variables such as immediate recall, free and cued recall after short delay, free and cued recall after long delay as well as recognition. A list of 16 words (four words of each category: fruit, clothing, drinks, tools) is read to the participant a total of five times (CVLT 1 … CVLT 5). After each round, the participant is encouraged to recall as many words as possible. Immediate recall is followed by a free and cued delayed recall after 5 (CLT short delay) and 20 min (CVLT long delay) intervals respectively, and a Yes/No recognition task (CVLT recognition).

##### Digit and Visual Span (Wechsler Memory Scale Revised, WMS-R; Härting et al., 2000)

The Digit Span test comprises digit span forward and digit span backward. In the digit span forward the participants are asked to repeat a sequence of digits until either the maximum number of eight digits per sequence is reached or until two consecutive incorrectly repeated sequences of same length. In the digit span backwards condition, the same procedure is applied with the task to repeat the digits backward. The same principle was implemented for the Visual Span using Corsi-block forward and backward. One point is given for each correct answer with scores ranging from 0 to 12 except for the forward visual span with scores ranging from 0 to 14.

##### Symbol Span (Wechsler Memory Scale—Fourth Edition, WMS-IV; Wechsler et al., 2012)

The Symbol Span subtest of the Wechsler Memory Scale (WMS)-IV assesses sequential working memory using abstract symbols as stimuli. The test requires recognizing previously presented symbols in their correct order. The number of symbols gradually increases from one to seven symbols, with each string of symbols being presented one after the other. After four consecutive errors, the test is terminated. Correctly recalled symbols in their right sequence are given 2 points, correct symbols in the wrong order are given 1 point, incorrectly recalled symbols are given 0 points.

##### Trail Making Tests A and B (TMT-A and TMT-B; Reitan and Wolfston, 1985)

The TMT are tests to assess visual attention and mental flexibility and requires an examinee to draw pencil lines in ascending order from 1 to 25 Trail Making Test A (TMT-A) and 25 encircled numbers and corresponding letters in an alternating order (TMT-B) that are randomly dispersed on a DIN-A-4 sheet. The discrepancy between the TMT-A and TMT-B (i.e., TMT-B minus TMT-A) is an indicator of deficits in mental flexibility. The instructions require working as fast as possible while maintaining maximum accuracy.

##### Fluency Tasks (Regensburg Verbal Fluency Test, RWT; Aschenbrenner et al., 2000)

RWT assesses semantic and phonetic verbal fluency. An examinee is instructed to generate as many words as possible in 1 min that belong to the category “animals” (semantic verbal fluency) as well as words starting with the letters “P” and “S” (phonemic fluency).

### Experimental Paradigms

News and commercials were used as a memory task representing the daily memory processes. To assess the relevance of this task all participants were asked whether or not they regularly watch news and commercials. Fourteen and 20 of YA and OA respectively, regularly watch news programmes. Likewise, seven YA and six OA reported to watch commercials.

#### News

Six news videos were shown to the participants with each video being between 27 s and 39 s. All videos were selected from the same popular daily TV news show *Tagesschau* from the ARD-channel and were originally broadcasted between the 1980s and early 1990s. We selected old news to avoid familiarity bias. The news topics pertained to domestic affairs (skateboard safety, merging of state-run train companies, changes in TV licence fee, river pollution, shortage in vocational training places, ferryboat incident). All six videos had the same format with a speaker (three female and three male speakers) and extra information such as a photo or a map being shown in the background.

#### Commercials

Six commercials were shown to the participants with each video being between 25 s and 34 s. All clips were selected from the internet and were originally broadcasted between the 1990s and early 2000s. The content of the commercials pertained to groceries of the brands that are still on the market today (beer, flour, rice, chocolate, detergent and grocery retailer).

#### Recognition Task

A recognition task with 12 statements was designed for each video to assess the number of correctly retrieved memory content from the respective video. The task for each video was presented immediately after watching the respective video. Six out of 12 statements contained information actually presented in the video (signal) whereas the remaining six statements contained information that was made up by the investigators to assess the number of erroneous memory items (noise). Out of the six signal statements asking about actually present information, three of them contained original information, and the other three were negated. There were three possible answer choices: “Yes” (the statement is true and directly corresponds to the video), “No” (the statement is true, but negated), and “Unknown” (the information has not been addressed or shown in the video). Three examples of such statements and the scoring system are presented in Table 1. In contrast to conventional recognition memory paradigms that rely on two answer choices (usually “old item” vs. “new item”), we have introduced negated statements to diminish older adult’s reliance on gist (Schacter et al., 1997; Tun et al., 1998; Dennis et al., 2007) by encouraging a more conscious decision making process, which has been reported to reduce the number of false alarms in older adults (Multhaup, 1995; Grady and Craik, 2000). The original German questionnaires administered to the patients together with the links to the videos used in the study are available from the corresponding author upon request.

**Table 1:** Scoring matrix for hits and false alarms used in the primary analysis.

			Answer choices		
Item type	Scene in film	Statement in questionnaire	Yes	No	Unknown
Original signal	The older woman^1^ offers rice^2^ to her granddaughter^3^	The older woman^1^ offers rice^2^ to her granddaughter^3^	**Correct** (hit)	Incorrect (miss)	Incorrect (miss)
Reversed signal	Skateboards^1^ are popular^2^ in Germany^3^	Skateboards^1^ are unpopular^2^* in Germany^3^	Incorrect (miss)	**Correct** (hit)	Incorrect (miss)
Noise	Not applicable	The woman^1^ wears a blue dress^2^ while baking^3^	Incorrect (false alarm)	Incorrect (false alarm)	**Correct** (correct rejection)

Superscript numbers mark separate features of a complex statement. Bold values mark correct responses. *Reversed feature of a recognition statement.

At the end of the experimental part, participants were asked to evaluate subjective feeling of difficulty of the questions on a 5-point Likert scale with “1” being very easy and “5” being very difficult as well as to give a subjective estimate of how many questions they answered correctly and how many questions were non-answerable.

### Procedure

Each participant completed a neuropsychological assessment prior to the experimental task. Before proceeding to the videos, participants were asked whether they watch news and commercials on a daily basis. Participants were then told that they will be shown short video clips of six news and six commercials, which will be followed a recognition task with three answer choices: (1) *yes, the statement is true*; (2) *no, the statement is false*; and (3) *unknown, the information from the statement was not presented in the video*. To illustrate the answer choices participants were given the following example: “Imagine that recognition statement says ‘*The apple in video was red*.’ If you remember seeing a red apple in the video, answer ‘*yes*.’ If you remember seeing a green apple in the video, answer ‘*no*.’ If you do not remember seeing an apple in the video at all, answer ‘*unknown*’.” After the instruction, participants proceeded to watch video clips. Recognition task for the respective video clip was presented immediately after the respective video. Answer choices were repeated prior to each recognition task. After watching all videos participants were asked to evaluate the perceived difficulty of the tasks and give an estimate of the number of correctly recognized items (hits) and correctly rejected false statements (correct rejections).

### Data Analysis

All statistical analyses were carried out using the SPSS (SPSS 21.0 for Windows, Chicago, IL, USA, 2012). The normality of distribution of hits and false alarms as well as the neuropsychological measures was tested with the Kolmogorov-Smirnov Test for each group separately. There was a homogeneity of variance between OA and YA for all the variables except MADRS, TMT-A, CVLT long delay, CVLT long-delay, CVLT hits, CVLT false alarms and subjective estimate of hits in commercials as assessed by Levene’s Test for Equality of Variances. In case of unequal variances, degrees of freedom were adjusted. Group comparisons were calculated using *t*-test, correlations using Pearson’s correlation coefficient. Other statistical procedures were used as indicated. Apart from comparing raw scores, the number of hits and false alarms was used to calculate the discriminability (d’) and bias (C) according to Signal Detection theory (Stanislaw and Todorov, 1999). Effect sizes were calculated using Cohen’s *d*.

## Results

There was a significant difference in the years of formal education among participants (YA 14.19 ± 2.14; OA 11.35 ± 2.60; *t*_(39)_ = 3.830, *p* < 0.001). However, education in older persons in Germany is just a weak indicator of general intellectual abilities due to the broken biographies in the sequels of WWII in Germany. Hence, the difference is representative of the official statistics within the German population (Statistisches Bundesamt, 2016). However, IQ scores for both groups were within a normal range (IQ YA 114.67 ± 9.04; IQ OA 107.90 ± 10.71). Demographic variables are shown in Table 2.

**Table 2:** Demographic and neuropsychological variables of young adults (YA) and older adults (OA).

	YA (*n* = 21)	OA (*n* = 20)			
	M ± SD	M ± SD	*t*	*p*	Cohen’s *d*
Age	26.90 ± 3.55	69.80 ± 5.85	−28.235	<0.001	8.87
MADRS	0.71 ± 1.35	2.55 ± 2.72	−2.715	0.011	0.86
MMSE	30 ± 0.00	29.65 ± 0.67	2.333	0.031	0.74
WST	35.10 ± 2.51	32.80 ± 3.55	2.401	0.021	0.75
DS forward	9.62 ± 1.53	8.70 ± 1.69	1.826	0.075	0.57
DS backward	8.43 ± 2.01	7.00 ± 2.45	2.044	0.048	0.64
VS forward	10.14 ± 2.24	7.00 ± 1.69	5.053	<0.001	1.58
VS backward	9.38 ± 1.75	6.40 ± 1.98	5.114	<0.001	1.59
Symbol test	27.95 ± 9.43	17.95 ± 8.36	3.586	0.001	1.12
TMT_A	19.86 ± 5.57	41.55 ± 11.56	−7.594	<0.001	2.39
TMT_B	47.57 ± 15.33	93.45 ± 30.36	−6.152	<0.001	1.91
TMT (B—A)	27.71 ± 13.56	51.90 ± 27.34	−3.616	<0.001	1.12
Semantic fluency	23.43 ± 4.90	24.50 ± 5.74	−0.644	0.523	0.20
Phonemic fluency “P”	12.43 ± 4.35	11.30 ± 3.39	0.923	0.362	0.29
Phonemic fluency “S”	16.05 ± 4.31	14.45 ± 3.76	1.262	0.214	0.40
CVLT_1	7.38 ± 1.69	5.10 ± 2.05	3.899	<0.001	1.21
CVLT_5	14.95 ± 1.43	12.70 ± 2.20	3.902	<0.001	1.21
CVLT_1_5	60.62 ± 8.75	47.35 ± 8.88	4.819	<0.001	1.50
CVLT_long_delay	14.14 ± 1.93	10.80 ± 2.84	4.387	<0.001	1.38
CVLT_hits	15.90 ± 0.30	15.55 ± 0.76	1.949	0.063	0.61
CVLT FA	0.10 ± 0.44	0.70 ± 1.49	−1.745	0.095	0.55

*Legend: CVLT, California Verbal Learning Test; DS, Digit Span; FA, false alarms; MADRS, Montgomery-Asberg-Depression-Rating-Scale; MMSE, Mini Mental State Examination; OA, older adults; TMT, Trail Making Test; VS, Visual Span; WST, vocabulary test (ger. Wortschatztest); YA, young adults*.

Older persons had significantly lower scores for the vocabulary test, the MMSE, and significantly higher scores for the MADRS. Despite the statistical significance, all scores for both groups were within a clinically normal range.

Older persons performed worse in measures of visual span and working memory, executive function (TMT), and most aspects of verbal memory than younger persons (Table 2). No group differences were observed in digit span as well as semantic and phonemic verbal fluency.

An overview of the number of times YA and OA answered *yes, no* or *unknown* for each item type (i.e., original signal, reversed signal, noise) is presented in Table 3.

**Table 3:** Means and Standard Deviations (M ± SD) for the number of responses given to each type of item.

		Given answer					
		*Yes*		*No*		*Unknown*	
Video Type	Item Type	YA	OA	YA	OA	YA	OA
News	Original signal (18 items)	**14.52 ± 1.57**	**13.40 ± 2.46**	1.52 ± 1.40	2.40 ± 1.35	0.90 ± 0.89	1.15 ± 1.60
	Reversed signal (18 items)	3.48 ± 1.60	4.05 ± 2.01	**13.86 ± 1.62**	**12.95 ± 1.57**	1.43 ± 1.29	1.45 ± 1.76
	Noise (36 items)	3.71 ± 1.93	5.90 ± 2.75	8.62 ± 4.31	8.70 ± 5.13	**23.38 ± 4.44**	**20.90 ± 6.32**
Commercials	Original signal (18 items)	**15.52 ± 1.78**	**14.35 ± 2.43**	1.95 ± 1.63	2.35 ± 1.63	0.52 ± 0.75	1.10 ± 1.65
	Reversed signal (18 items)	2.52 ± 1.40	3.30 ± 2.11	**13.95 ± 1.75**	**11.80 ± 2.69**	1.38 ± 1.12	2.50 ± 2.65
	Noise (36 items)	4.76 ± 3.79	4.45 ± 2.28	6.05 ± 3.01	7.35 ± 4.57	**24.57 ± 5.35**	**23.45 ± 5.04**

*Legend: OA, older adults; YA, young adults. Bold values mark correct responses*.

Before calculating the group differences in recognition, we compared the subjective difficulty, subjective performance estimate and the news and commercials watching habits between the two groups (Table 4). While potential group differences of material relevance between YA and OA were not directly measured, we measured frequency of watching news and commercials and subjective difficulty. Our results show that OA watch news significantly more often ($\chi_{\left(1\right)}^{2}$ = 8.039, *p* = 0.005) and subjectively perceived questions to the news material as significantly easier than YA (YA 3.48 ± 0.81, OA 2.84 ± 0.90; *t*_(38)_ = 2.343, *p* = 0.024, effect size 0.84). Although there was no significant difference in the frequency of watching advertisements between the two groups ($\chi_{\left(1\right)}^{2}$ = 0.053, *p* = 0.819), OA perceived the questions accompanying the advertisement videos as being significantly harder (YA 2.71 ± 0.72, OA 3.26 ± 0.87; *t*_(38)_ = −2.183, *p* = 0.035, effect size 0.69).

**Table 4:** Hits and false alarms on watching news and commercials in young adults (YA) and older adults (OA).

	YA (*n* = 21)	OA (*n* = 20)
	M ± SD	M ± SD	*t*	*p*	Cohen’s *d*
**News**					
Hits^1^	28.38 ± 2.27	26.40 ± 3.07	2.359	0.023	0.73
FA^2^	12.33 ± 4.45	14.55 ± 6.22	−1.317	0.195	0.41
Subjective Difficulty^3^	3.48 ± 0.81	2.84 ± 0.90	2.343	0.024	0.75
Hits subjective^4^	43.71 ± 11.80	43.56 ± 13.47	0.035	0.972	0.01
FA subjective^4^	20.29 ± 8.09	19.17 ± 6.99	0.419	0.678	0.15
**Commercials**
Hits^1^	29.33 ± 2.82	26.10 ± 3.24	3.413	0.002	1.06
FA^2^	10.76 ± 4.88	11.85 ± 4.64	−0.731	0.469	0.23
Subjective Difficulty^3^	2.71 ± 0.72	3.26 ± 0.87	−2.183	0.035	0.69
Hits subjective^4^	48.50 ± 10.43	39.61 ± 18.05	1.748	0.091	0.60
FA subjective^4^	21.14 ± 8.38	18.17 ± 8.32	1.001	0.325	0.36

*Legend: FA, false alarms; OA, older adults; YA, young adults. ^1^Hits as a sum of correct answers to original and reversed statements (see Table 1), total of 36 items; ^2^Total of 36 items; ^3^Difficulty measured on a 5-Point Likert Scale; ^4^Participants’ subjective estimates of the number of correct responses (hits and correct rejections) and false alarms out of 72 items*.

To control for the effect of commercials watching habits on the recognition outcome, we conducted a 2 (YA vs. OA) × 2 (watching commercials vs. not watching commercials) ANOVA with hits in commercials recognition task as a dependent variable. There was no significant interaction between the effects of age and commercials watching habits, *F*_(1,37)_ = 0.345, *p* = 0.561. There was a significant main effect of age group *F*_(1,37)_ = 8.619, *p* = 0.006; however not of the commercials watching habits *F*_(1,37)_ = 0.526, *p* = 0.473. The same procedure was repeated with false alarms for commercials as a dependent variable. There was no significant interaction between age and commercials watching habits *F*_(1,37)_ = 0.325, *p* = 0.572 and no significant main effect of either age (*F*_(1,37)_ = 0.192, *p* = 0.664) or watching habits (*F*_(1,37)_ = 0.377, *p* = 0.543) on false alarms. As all of the participants claimed to watch news regularly, our data did not allow us to look into the same effects for news; however, we assume that the influence of news and commercials watching habits on recognition memory works similarly in news as in commercials.

An overview of subjective and objective hits, false alarms is presented in Table 4. Subjective estimation of number of hits and correct rejections was comparable in YA and OA. YA had significantly more hits in the videos than OA in both, news and commercials. In contrast, the number of false alarms was similar in OA and YA.

Analysis of discriminability has revealed a significant difference in d′ between YA (1.26 ± 0.46) and OA (0.89 ± 0.55), *t*_(39)_ = 2.339, *p* = 0.025 (effect size 0.73) in news and commercials recognition task, *t*_(39)_ = 2.821, *p* = 0.007 (YA 1.49 ± 0.46; OA 1.08 ± 0.48; effect size 0.87).

Both OA and YA displayed a liberal answering strategy with mean C values being below zero for both groups in news (YA −0.19 ± 0.21; OA −0.20 ± 0.30, effect size 0.04) and in commercials (YA −0.19 ± 0.26; OA −0.07 ± 0.21, effect size 0.50). The groups showed no differences in bias, *t*_(39)_ = 0.086, *p* = 0.932 and *t*_(39)_ = −1.505, *p* = 0.140 for news and commercials, respectively.

Contrary to our hypothesis, OA and YA had a similar amount of false alarms in both paradigms. However, in line with the literature (e.g., Fraundorf et al., 2019), OA had significantly fewer hits and inferior discriminability in both news and commercials paradigm.

As our paradigm represents a modified use of a signal detection theory by incorporating reversed signal items, we wanted to rule out gist influence on older adults and ran an alternative analysis by dividing the signal items into original statements and reversed statements. Koutstaal and Schacter (1997) and Schacter et al. (1999) described a procedure in which in addition to classical signal detection theory (comparison of signal vs. noise) noise items are compared to items strongly related to original signal items. This procedure was developed to assess false recognition of lure items conceptually, perceptually, or semantically related to studied items and aims to draw a distinction between baseline false alarms and memory mistakes as a result of similarity to signal items or gist memory.

According to this modification of signal detection theory we first compared discriminability and bias measures between the YA and OA using *yes* answers to original signal items (hits) to the sum of *yes* and *no* answers to noise items (false alarms) to evaluate a baseline false alarm rate. This analysis omitted hits to reversed items. The alternative scoring system is displayed in Table 5. There were no significant differences for the number of hits to original signal items between the groups (news: YA 14.52 ± 1.57, OA 13.40 ± 2.46, effect size 0.54, *t*_(39)_ = 1.754, *p* = 0.087; commercials: YA 15.52 ± 1.78, OA 14.35 ± 2.43, effect size 0.55, *t*_(39)_ = 1.770, *p* = 0.085). There was a significant difference in discriminability for news (YA 1.56 ± 0.57, OA 1.13 ± 0.56, effect size 0.65, *t*_(39)_ = 2.465, *p* = 0.018) but not for commercials (YA 1.75 ± 0.59, OA 1.41 ± 0.74, effect size 0.73, *t*_(39)_ = 1.624, *p* = 0.112). There were no differences in bias (news: YA −0.35 ± 0.29, OA −0.32 ± 0.42, effect size 0.08, *t*_(39)_ = 2.465, *p* = 0.018; commercials: YA −0.31 ± 0.30, OA −0.24 ± 0.28, effect size 0.24, *t*_(39)_ = −0.834, *p* = 0.409).

**Table 5:** Scoring matrix for hits and false alarms used in the secondary analysis without the reversed signal items.

			Answer choices		
Item type	Scene in film	Statement in questionnaire	*Yes*	*No*	*Unknown*
Original signal	The older woman^1^ offers rice^2^ to her granddaughter^3^	The older woman^1^ offers rice^2^ to her granddaughter^3^	Hit	Miss	Miss
Noise	Not applicable	The woman^1^ wears a blue dress^2^ while baking^3^	False alarm	False alarm	Correct rejection

*Superscript numbers mark separate features of a complex statement*.

Similarly, we next compared discriminability and bias using *yes* answers to reversed signal items (hits for gist signal) and false alarms to assess gist memory. This analysis omits the original signal items. The scoring system designed to measure the strength of the gist signal is displayed in Table 6. There were no significant differences in the hits to gist signal between OA and YA (news: YA 3.52 ± 1.54, OA 4.35 ± 1.81, effect size 0.49, *t*_(39)_ = −1.576, *p* = 0.123, commercials: YA 2.48 ± 1.44, OA 3.35 ± 1.95, effect size 0.50, *t*_(39)_ = −1.637, *p* = 0.110). There were also no group differences in either discriminability (news: YA −0.49 ± 0.38, OA −0.47 ± 0.48, effect size 0.05, *t*_(39)_ = −0.181, *p* = 0.858; commercials: YA −0.59 ± 0.65, OA −0.51 ± 0.58, effect size 0.13, *t*_(39)_ = −0.427, *p* = 0.671) or bias (news: YA 0.68 ± 0.31, OA 0.48 ± 0.36, effect size 0.60, *t*_(39)_ = 1.896, *p* = 0.065; commercials: YA 0.86 ± 0.23, OA 0.72 ± 0.30, effect size 0.52, *t*_(39)_ = 1.624, *p* = 0.112) for gist memory.

**Table 6:** Scoring matrix for hits and false alarms used to assess sensitivity to gist signal.

			Answer choices		
Item type	Scene in film	Statement in questionnaire	*Yes*	*No*	*Unknown*
Gist signal	Skateboards^1^ are popular^2^ in Germany^3^	Skateboards^1^ are unpopular^2^* in Germany^3^	Hit	Miss	Miss
Noise	Not applicable	The woman^1^ wears a blue dress^2^ while baking^3^	False alarm	False alarm	Correct rejection

Superscript numbers mark separate features of a complex statement. *Reversed feature of a recognition statement.

## Discussion

It is generally acknowledged that memory performance decreases with age. Research on age-associated memory deficits explored different aspects of both recall and recognition. The majority of the paradigms in such studies employed stimuli that are only indirectly related to everyday life. The present study employed a type of stimuli that younger and older adults encounter on a daily basis. Participants were shown news and commercials and subsequently performed a recognition task to assess hits and false alarms for video content.

News and commercials watching habits did not have an effect on the recognition task outcome. Older adults subjectively found news recognition task easier, whereas young adults found commercials recognition task easier. Despite the differences in the subjective difficulty, there was no difference between the two groups in the subjective estimate of correct responses. This result helps to exclude possible adverse effects due to low performance expectations among older adults (Chasteen et al., 2005; Hess and Hinson, 2006; Hess et al., 2009). Contrary to our hypothesis, the older adults did not have more false alarms compared to young adults, despite younger adults scoring significantly better in most of the neuropsychological variables. There was, however, a difference in the number of hits, with younger adults performing significantly better in both experimental paradigms. After calculating the effect size, we observed that older adults scored slightly over one half of a standard deviation worse on news hits and one standard deviation worse on commercials hits. Signal detection theory supports these results. Discriminability scores for news and commercials in young adults were significantly higher. Discriminability is a measure calculated using both hits and false alarms. Group variance of hits and group variance of false alarms was comparable. Thus, the observed discriminability difference results from the difference in the number of hits. Older and younger adults showed no differences regarding answering tendencies and both have shown moderately liberal bias. There have been some reports, that response bias might be a stable cognitive trait (Kantner and Lindsay, 2012, 2014) and that the decision criterion shifts as a function of memory strength, strategy, personality and affect (Aminoff et al., 2012).

As our hypothesis on increased false alarms in older adults was rejected, we could not further pursue the original plan of looking into what neuropsychological variables explain differences in false alarms between young and older adults. Nevertheless, the outcomes of neuropsychological assessment are in harmony with age-related cognitive deficits reported in the literature (Ardila et al., 2000; Park et al., 2002; Zahodne et al., 2011; Lipnicki et al., 2013). The present study observed a generally diminished episodic memory performance in older persons. Differences were observed only in the tasks of free recall, and not recognition memory, which most likely represent a ceiling effect in the test’s sensitivity. Moreover, attention, working memory as well as some aspects of executive function (as measured by the Trail Making Test) were diminished. Other measures of executive function (semantic and phonemic verbal fluency) did not differ in younger and older persons. None of the deficits in older adults were clinically indicative of pathology. This significantly reduces the possibility that the failure to demonstrate age-related increase in false alarms was due to unusually high cognitive performance in this sample of older adults.

The present study supports earlier findings of aging-related decrease in hits, however, it contradicts reports on age-associated increase in false alarms (Seamon et al., 2002; Dennis et al., 2008; Rosa and Gutchess, 2013; Devitt and Schacter, 2016; Paige et al., 2016). Out of the three recognition memory theories described in the introduction, the associative deficit hypothesis (Naveh-Benjamin, 2000; Old and Naveh-Benjamin, 2008) offers a possible explanation for the absence of an increase in false alarms among older adults in our study. Chalfonte and Johnson (1996) argued that association (binding) of features belonging to the same stimulus enriches memory. Hence, what we remember is not *blue* and *pen* but rather *blue pen* as a single entity.

The results of the current study can be interpreted in a similar way. In order to make a decision about the truthfulness of a statement in our paradigm, participants were required not only to memorize but also to bind the features together into a single entity. In order to correctly accept a correct statement [*The older woman*^1^ *offers rice*^2^ *to a girl*^3^], one needs to have encoded and bound all three features correctly (square brackets indicate that the features form a single signal). The recognition of some item features is not enough for a hit, as our paradigm introduces ambiguity, where half of the original items are presented in the negated form. The recognition process for the negated form of the original signal is similar: a composite item [*Skateboards*^1^ *are popular*^2^ *in Germany*^3^] needs to be bound and encoded as a single entity in order to recognize that the statement presented in the recognition task, [*Skateboards are unpopular in Germany*], is the negated form of the original statement. Any information unit missing or being different negates the original message signal in a Boolean sense and should induce a “no” in recognition of this composite item. Since the item is treated as one homogeneous signal it needs to be recognized or negated in its entirety. If it is not recognized completely, it is missed: remembering only *skateboards* or only *Germany* is not enough for a successful recognition. In contrast, items that present noise without any elements of a signal, e.g., [*The woman*^1^ *wears a blue dress*^2^ *while baking*^3^] should only be answered with “unknown,” as in the whole videos sequence there is no [*Woman wearing a blue dress while baking*]. Anything but the response “unknown” represents a false alarm.

In their meta-analysis, Fraundorf et al. (2019) reported that young adults mostly outperform older adults in easy recognition tasks but in some instances of more complex recognition tasks, older adults performed at least as good as or better than young adults. The authors were not able to identify any variables that would explain preserved memory performance in older adults in their meta-analysis. However, this special circumstance seems to appear in studies using ecologically valid tasks with complex recognition statements (LaVoie and Malmstrom, 1998; Matzen and Benjamin, 2013). The present study offers additional support to this possibility and is in-line with a repeatedly documented decrease in binding ability among older adults (Chalfonte and Johnson, 1996; Naveh-Benjamin, 2000; Old and Naveh-Benjamin, 2008; Fraundorf et al., 2019). While making a correct recognition harder due to the binding deficit, complex items might paradoxically offer an advantage to older adults, when it comes to correct rejection, because several false features within a noise item make this item more salient and easier to identify as noise.

After separating original and reversed statements in the subsequent analysis we have found that statistically significant difference in hits between the groups has disappeared, however, the trend has remained. It could be that statistical significance was affected by the decrease in items after separating original and reversed signal items. Alternatively it could be speculated that inferior performance of older adults in recognition task for reversed statements might result from their reduced cognitive processing abilities, as processing of sentential negation requires additional neural resources of response inhibition to the original affirmative form of the particular item (Tettamanti et al., 2008; Bartoli et al., 2013; Beltrán et al., 2018). Furthermore, using the same analysis the difference in the discriminability for commercials disappears. This might indicate slight differences in “news” stimuli compared to “commercials” stimuli, which needs to be addressed in future studies. Further studies are needed to address these issues.

Calculating susceptibility to gist memory by examining discriminability and bias estimates calculated from yes responses to reversed signal items showed no group differences. Although previous studies have reported a greater reliance on gist among older adults (e.g., Koutstaal et al., 1999; LaVoie and Faulkner, 2000), the failure to observe it in our study could be attributed to several factors. The participants in our study were given detailed information about the items they will be confronted with in the recognition task and what type of answer is appropriate for each item. Such *a priori* instructions and warnings have been shown to decrease memory errors in older adults (Carmichael and Gutchess, 2016). It is also possible that the timing of the recognition task has contributed to a decreased reliance on gist. The recognition task in our study was performed right after seeing the actual material, which means that the verbatim traces were still robustly represented in the participant’s memory and the reliance on gist, which increases with the temporal deterioration of the verbatim traces, was not yet necessary (Abadie and Camos, 2018). It is important to note that despite susceptibility to gist signal not providing a statistically significant group difference, it showed a moderate effect size. This suggests that this result might reflect a lack of power rather than a genuine absence of performance differences between young and older adults.

There are several limitations to the study. First, demographic variables for general intellectual abilities, overall cognitive score, and assessment of mood were different between young and older persons. The differences were small and all scores in both groups were within normal range and representative of the general population, yet it cannot be excluded that it had an influence on the results. Moreover, the relevance of the information reported in the news clips and the familiarity with the products mentioned in the commercials was not assessed. It was previously reported that factors such as selectivity in task engagement and perceived emotional goals affect memory performance in the older adults (Hess, 2005), thus we cannot rule out that these differences exist between or within the age groups. Future paradigms addressing ecologically valid memory tasks should further explore the effects of familiarity and the perceived meaningfulness of the task.

## Conclusion

Decrease in correct recognitions and increase in false alarms among older adults has been well documented and explained by deficits in feature binding, inhibition and cognitive slowing. The present study demonstrated that age-related increase in false alarms is not universal but subject to the complexity of the stimuli. An ecologically valid task requiring binding of several features increases the likelihood of a correct rejection. This demonstrates that while memory is patchy in older persons it is not distorted for real-life situations.

## Ethics Statement

The study received approval of the local ethics committee of the Ulm University (Application No. 233/15). All persons gave their informed consent prior to their participation in the study.

## Author Contributions

KS and CL were involved in acquisition of the data, data analysis, and drafting and revising the manuscript. KS, CL and MR were involved in designing the study, interpretation of the data, and drafting and revising the manuscript. All authors approved the final version of the manuscript.

## Conflict of Interest Statement

The authors declare that the research was conducted in the absence of any commercial or financial relationships that could be construed as a potential conflict of interest.

## Abbreviations

CVLT: California Verbal Learning Test
DRM: Deese-Roediger-McDermott procedure
FA: False alarms
MADRS: Montgomery-Asberg-Depression-Rating-Scale
MMSE: Mini Mental State Examination
OA: Older adults
RWT: Regensburg Verbal Fluency Test
TMT: Trail Making Test
WMS: Wechsler Memory Scale
WST: Vocabulary test
YA: Young adults.
